# TARGET: A Singapore National Cohort for Assessing Geriatric Fall and Fracture Risk in the Community

**DOI:** 10.1093/geroni/igaf122.980

**Published:** 2025-12-31

**Authors:** Kok Yang Tan, Angelique Chan, Rahul Malhotra, Abhijit Visaria, Vanessa Koh, Lakkhina Troeung, David Matchar, William Taylor

**Affiliations:** Duke-NUS Medical School, Singapore, Singapore; Duke-NUS Medical School, Singapore, Singapore; Duke-NUS Medical School, Singapore, Singapore; Duke-NUS Medical School, Singapore, Singapore; Duke-NUS Medical School, Singapore, Singapore; Duke-NUS Medical School, Singapore, Singapore; Duke-NUS Medical School, Singapore, Singapore; Institute for Biomechanics ETH Zürich, Zürich, Zurich, Switzerland

## Abstract

The Targeted Assessment and Recruitment of Geriatrics for Effective fall prevention Treatments (TARGET) study is a national prospective cohort study in Singapore, integrating novel health technology approaches, to develop cost-effective and scalable frameworks for early detection and prevention of falls and fractures in community-dwelling older adults. TARGET combines epidemiological survey data with data from novel wearable sensor, clinical imaging and virtual reality (VR) technology to predict the risk of incident falls and hip fractures. Between October 2022 and October 2024, a total of 2,291 Singapore residents aged 60 years and older were enrolled. Participants underwent a baseline fall risk assessment (T0) comprising (i) an interview to capture sociodemographic, anthropometric, physical, functional, and psychosocial measures, (ii) gait assessment using wearable inertial measurement unit motion sensors, (iii) dual-energy X-ray absorptiometry and whole-body 3D scans, and (iv) VR-based spatial navigation assessment. While all participants underwent (i) and (ii), a subset (13%-39%) underwent (iii) to (v). Prospective follow-up of participants – every three months for two years (T1-T8) to monitor incident falls, fractures and cognitive status – is ongoing. Electronic medical records are also being linked, providing objective longitudinal data on fall-related morbidity, mortality, healthcare utilisation and costs. Statistical analyses focus on (i) developing and validating personalised risk prediction models to identify fallers, (ii) developing hip fracture risk prediction models for clinical decision-making, (iii) assessing the predictive utility of VR technology for detecting cognitive decline and fall risk, and (iv) understanding the psychological and sociodemographic correlates of fall risk in older adults.

